# On the Convergence of the Number of Exceedances of Nonstationary Normal Sequences

**DOI:** 10.6028/jres.099.051

**Published:** 1994

**Authors:** J. Hüsler, M. Kratz

**Affiliations:** University of Bern, Bern, Switzerland; University Paris VI, Paris, France

**Keywords:** exceedances, nonstationary, normal sequences, rate of convergence, Stein-Chen method

## Abstract

It is known that the number of exceedances of normal sequences is asymptotically a Poisson random variable, under certain restrictions. We analyze the rate of convergence to the Poisson limit and extend the result known in the stationary case to nonstationary normal sequences by using the Stein-Chen method. In addition, we consider the cases of exceedances of a constant level as well as of a particular nonconstant level.

## 1. Introduction and Result

The extreme value theory of Gaussian sequences has interested many authors, for instance Refs. [1,4,7,8], dealing with the limit distribution of the suitably normalized extreme value.

Let {*X_i_*,*i* ≥ 1} be a standardized normal sequence with correlations *E*(*X_i_X_j_*) = *r_ij_*,*i*,*j* ≥ 1, and *Φ*(·) the distribution function of *X_i_.*

Let

$$N_{n}=\sum_{i=l}^{n}1(X_{i}>u_{ni})$$

denote the number of exceedances of a boundary given by a triangular array {*u_ni_*,*i* ≤ *n*,*n* ≥ 1}. Then it was also found that *N_n_* converges in distribution to a random variable having a Poisson distribution *ℙ*(λ*_n_*), if the mean number of exceedances $\lambda_{n}=\sum_{i\leq n}\left(1-\Phi \left(u_{ni}\right)\right)$ remains bounded (cf. Ref. [4]).

For practical use of the asymptotic theory, it is rather important to know the rate of convergence or at least some upper bound for this rate.

For the stationary case, results on the rate of convergence have been obtained for instance by Refs. [2,3,9,10]. The aim of this paper is to give an upper bound for the total variation distance *d_IV_*. between *N_n_* and *ℙ*(λ*_n_*), in the nonstationary case, extending the results of these mentioned papers.

Suppose that for some sequence *p_n_*: |*r_ij_*| ≤ *pi* − *j*| for (*i* ≠ *j* and that the two conditions

$$\rho_{n}\;<\;1\;\text{for}\;\text{all}\;n\geq 1$$ (1)

$$\rho_{k}\leq A/\log\;k,k\geq 2,\;\text{for}\;\text{some}\;\text{constant}\;A$$ (2)

are satisfied. Define *p* as *p* = max(0, *r_ij_*, *i* ≠ *j*) < 1.

In addition, we assume that the boundary values tend uniformly to ∞:

$$u_{n,\min}=\underset{l\;\leq \;i\;\leq \;n}{\min}\;u_{ni}\;\to \;\infty \;\text{as}\;n\to \;\infty .$$ (3)

The exceedances of a constant boundary *u_ni_* = *u_n_*, 1 ≤ *i* ≤ *n*, are considered first, where only the tools given in Ref. [3] are used. We show in the second result that the method of Ref. [3] can be used also for nonconstant boundaries {*u_ni_*}. But these boundaries are restricted such that the condition

$$\underset{n\;\to \;\infty }{\lim\;\sup}n\;(1-\Phi (u_{n,\min}))<C<\infty$$ (4)

holds. If we want to extend the results to a more general class of boundaries such that only

$$\underset{n\;\to \;\infty }{\lim\;\sup}\;\lambda_{n}\;<\;C\;<\infty$$ (5)

holds, we need to combine the method developed by Ref. [4] with that of Ref. [3] to get satisfactory results (see Ref. [6]).

Our first result for boundaries which are constant for fixed *n*, shows that the given upper bound of the rate of convergence depends mainly on the largest positive correlation value *ρ*.

**Theorem 1:***Let* {*X_i_*,*i* ≥ 1} *be a standardized nonsta-tionary normal sequence with correlations {r_ij_*, *i*, *j* ≥ 1}. *Suppose that |r_ij_*| ≥ *p_|i_*_−_ *_j|_ for i* ≠ *j*, *such that Eqs. (1) and (2) hold. Let the boundary values* {*u_ni_* − *u_n_*,1 ≤ *i* ≤ *n*} *and* λ*_n_ he real values with* λ*_n_* = *n*(1 − *Φ*(*u_n_*)). *Suppose that* λ*_n_* ≤ *C* < ∞, *for some constant C. Then as n* → *∞*

$$\begin{array}{c} d_{n}(N_{n},\mathbb{P}(\lambda_{n}))=O(n{\;}^{-\frac{1-\rho }{1+\rho }}\cdot (\log\;n{)}^{-\frac{\rho }{1+\rho }}\\ +\frac{\log\;n}{n^{2}}\sum_{k=1}^{n-1}(n-k)\rho_{k}). \end{array}$$ (6)

This extends the result of Ref. [3] showing that for a constant boundary their upper bound of the rate of convergence in the stationary case holds also in the nonstationary case.

**Theorem 2:***Let* {*X_i_*,*i* ≥ 1} *be a standardized nonstationary normal sequence with correlations* {*r_ij_*, *i*,*j* ≥ 1} *as in Theorem 1 satisfying Eqs. (1) and (2). Suppose that the boundary values* {*u_ni_*, 1 ≤ *i* ≤ *n*,*n* ≥ 1} *are such that* Eq. (4) *holds*, *Then* Eq. (6) *holds.*

The first term of Eq. (6) dominates the rate of convergence in cases with *∑_k_*_≥ 1_ *ρ_k_* < *∞* and *ρ* > 0.

Then the rate of convergence depends only on the lowest value *u_n_*_,min_ of the particular boundary *u_ni_* and also on the largest positive correlation *p.* It extends naturally the results of the stationary case with boundary values which are constant for fixed *n.* This rate is only good if *u_n_*_,min_ is not a uniquely low value, which is supposed for reasonable boundaries. For the case *u_n_*_,min_ is uniquely low, the rate can be improved.

## 2. Proof

The proof of Theorem 1 is an adaption of that used by Ref. [3] in the stationary case. We use the following lemma which is a straightforward extended version of Lemma 3.4 of Ref. [3].

**Lemma 1:***Suppose that*

$$(X_{i},X_{j})\overset{d}{=}N\left(\left(\begin{array}{c} 0\\ 0 \end{array}\right),\left(\begin{array}{c} 1\\ r_{ij}\; \end{array}\begin{array}{c} r_{ij}\\ 1 \end{array}\right)\right).$$

*Define Z_i_* = 1(*X_i_* > *u_ni_*) *for some boundary values u_ni_ where*

$$1-\Phi (u_{ni})\leq \;C\;/n$$

*for some finite constant C.*

*Then for some constant K depending on C only and for all n* ≥ 2:

- *If* 0 ≤ *r_ij_* < 1, *then*
  $$\begin{array}{c} 0\leq \;\mathrm{cov}(Z_{i},Z_{j})\leq \;K\;\cdot \frac{1}{\sqrt{1-r_{ij}}}\;n^{-2+\frac{2r_{ij}}{1+r_{ij}}}(\log\;n{)}^{-\frac{r_{ij}}{1+r_{ij}}}\\ \leq K\;n{\;}^{-2}(\frac{n^{2}}{\log\;n}{)}^{\frac{\rho_{ij-i1}}{1+\;\rho_{i-j}|}} \end{array}$$
- *If* 0 ≤ *r_ij_* ≤ 1, *then*
  $$\begin{array}{c} 0\leq cov(Z_{i},Z_{j})\leq K\frac{r_{ij}\;\log\;n}{n^{2}}e^{2r_{ij}\log\;n}\\ \leq K\frac{\rho_{i-ji}\log\;n}{n^{2}}\;e^{2_{p1-ji}\log\;n} \end{array}$$
- *If* − 1 < *r_ij_* ≤ 0, *then*
  $$0\geq cov\;(Z_{i},Z_{j})\geq -K\frac{|r_{ij}|\log\;n}{n^{2}}\geq -K\frac{\rho_{\left|j-j\right|}\log\;n}{n^{2}}$$
- *If* − 1 ≤ *r_ij_* ≤ 0, *then*
  $$0\geq cov(Z_{i},Z_{j})\geq -K\frac{1}{n^{2}}$$

We need for Theorem 2 an extension of Lemma 1.

**Lemma 2:***Suppose that* (*X_i_*,*X_j_*) *and Z*, *are as in Lemma 1. For any i*,*j*, *define u_nij_* = min (*u_ni_*, *u_nj_*) *and v_nij_* = max(*u_ni_*,*u_nj_*).

*Then for some constant K depending only on C and for all n* ≥ 2:

- *i) If* 0 ≤ *r_ij_* ≤ 1, *then*
  $$\begin{array}{l} 0\leq cov\;(Z_{i},Z_{j})\leq K\frac{1}{\sqrt{1-r_{ij}}}{\left(\frac{\varphi (u_{nij})}{u_{nij}}\right)}^{\frac{2}{1+r_{ij}}}\cdot u_{nij}^{-\frac{2r_{ij}}{1+r_{ij}}}\\ with\;K\geq (2\text{π)}{\;}^{-\frac{r_{ij}}{1+r_{ij}}}\cdot {\left(1+r_{ij}\right)}^{3/2}. \end{array}$$
- *If* 0 ≤ *r_ij_* ≤ 1, *then*
  $$0\leq cov\left(Z_{i},Z_{j}\right)\leq K\;r_{ij}\;\varphi \left(u_{nij}\right){\left(\varphi (v_{nij})\right)}^{\frac{1-r_{ij}}{1+r_{ij}}}$$
- *If* − 1 ≤ *r_ij_* ≤ 0, *then*
  $$\begin{array}{c} 0\geq cov\left(Z_{i},Z_{j}\right)\geq -\left(1-\Phi \left(u_{nij}\right)\right)\left(1-\Phi \left(v_{nij}\right)\right)\\ \geq -{\left(1-\Phi \left(u_{nij}\right)\right)}^{2} \end{array}$$
- *If* − 1 ≤ *r_ij_* ≤ 0, *then*
  $$\begin{array}{c} 0\leq \;\text{|}cov\left(Z_{i},Z_{j}\right)\text{|}\;\leq K\;\min\left(1\text{,|}r_{ij}\text{|}u_{nij}v_{nij}+r_{ij}^{2}v_{nij}^{2}\right)\\ \left(1-\Phi \left(u_{nij}\right)\right)\cdot \left(1-\Phi \left(v_{nij}\right)\right) \end{array}$$

(The proof of this lemma is given in [6]).

Theorem 1 follows also by Theorem 2. Therefore, we prove now Theorem 2 by using the method of Ref. [3].

By Theorem 3.1 of Ref. [3], we have

$$d_{iv}\left(N_{n},\mathbb{P}\left(\lambda_{n}\right)\right)\leq \frac{1-e^{-\lambda_{n}}}{\lambda_{n}}\left(\frac{\lambda_{n}^{2}}{n}+\sum_{ixj}\left|cov\left(Z_{i},Z_{j}\right)\right|\right).$$ (7)

If *p* = 0, i.e. if *r_ij_* ≤ 0 for *i* ≠ *j*, then using Lemma 2(iv) for the second term of Eq. (7), we get the second term of Eq. (6) which dominates the first one in this case, if *ρ_k_* > 0 for some *k.* Obviously, if *ρ_k_* = 0 for all *k*, then the result holds, since the second term in Eq. (7) is 0.

Thus suppose from now on that *p* > 0.

Because of Eq. (2) the sum

$$S_{n}=\sum_{1\leq i\;<j\leq n}|cov\left(Z_{i},Z_{j}\right)\text{|}\;\text{=}\;\sum_{1\leq i<j\leq n}c_{ij}$$

is split up into three parts, by using δ > 0 such that

$$3\delta <\frac{\rho }{1+\rho }$$

There are only finitely many *k’*s with *ρ_k_* > δ. This will be treated first as Case (i). For indices *k* with *ρ_k_* ≤ δ, we distinguish Case (ii) with *k < n*^δ^ and Case (iii) with *k* ≥ *n*^δ^.

Case (i): Each term *c_ij_* of the sum *S_n_* is bounded above by

$$K\;n^{-2/(1+\rho )}(\log\;n{)}^{-\rho /\left(1+\rho \right)}$$

if *r_ij_* ≥ 0 by Lemma 2(i) or bounded by

$$K\;n^{-2}$$

if *r_ij_* < 0 by Lemma 2(iii).

Since there are finitely many *k*’s with *ρ_k_* > δ, the number of terms *c_ij_* with |*i* − *j*| = *k* and *ρ_k_* > δ is of the order O(*n*). Hence the sum on these terms is bounded by

$$K\;n^{-\left(1-\rho \right)/\left(1+\rho \right)}{\left(\log\;n\right)}^{-\rho /\left(1+\rho \right)}+K\;n^{-1}.$$

Case (ii): There are at most *n*^δ^ terms *ρ_k_* such that 0 ≤ *ρ_k_* ≤ δ and *k* < *n*^δ^. Hence there are at most O(*n*^1 + δ^) terms *c_ij_* with such a *k* = |*i* − *j*|. But each such term *c_ij_* of *S_n_* is bounded by

$$K\;n^{-2/\left(1+\delta \right)}{\left(\log\;n\right)}^{-\delta /\left(1+\delta \right)}\leq K\;n^{-2+2\delta }$$

if *r_ij_* ≥ 0 by Lemma 2(i) or bounded by

$$K\;n^{-2}$$

if *r_ij_* < 0 by Lemma 2(iv).

Then the sum of these terms *c_ij_* is bounded by *O*(*n*^−1 + 3δ^).

Case (iii): Finally we consider the terms such that *ρ_k_* ≤ δ, with *k* ≥ *n*^δ^. Note that we have

$$0\leq \rho_{k}\leq \frac{A}{\log\;k}\leq \frac{A}{\delta \log\;n}.$$ (8)

Each such term *c_ij_* of *S_n_* gives a contribution

$$K\;\rho_{k}{\left(\frac{\sqrt{\log\;n}}{n}\right)}^{1+\frac{1-\rho_{k}}{1+\rho_{k}}}\leq K\;\rho_{k}\frac{\log\;n}{n^{2}}$$

if *r_ij_* ≥ 0 by Lemma 2(ii) and by using Eq. (8)

$$K\;\rho_{k}\left(\frac{\log\;n}{n^{2}}\right)$$

if *r_ij_* < 0 by Lemma 2(iv).

Taking now the sum on all terms (*i*,*j*) with |*i* – *j*| ≥ *n*^δ^, we get the second term of Eq. (6).

Finally, adding up all these upper bounds of Cases (i), (ii), and (iii), the result Eq. (6) of Theorem 2 follows.
